# Effects of Adding an Online Exercise Program on Physical Function in Individuals Hospitalized by COVID-19: A Randomized Controlled Trial

**DOI:** 10.3390/ijerph192416619

**Published:** 2022-12-10

**Authors:** Luis Llurda-Almuzara, Jacobo Rodríguez-Sanz, Carlos López-de-Celis, Ramón Aiguadé-Aiguadé, Raúl Arán-Jové, Noé Labata-Lezaun, César Fernández-de-las-Peñas, Joan Bosch, Albert Pérez-Bellmunt

**Affiliations:** 1 Physiotherapy Department, Faculty of Health Sciences, European University of Gasteiz—EUNEIZ, La Biosfera Ibilbidea, 6, 01013 Vitoria-Gasteiz, Spain; 2 Faculty of Medicine and Health Sciences, Universitat Internacional de Catalunya (UIC-Barcelona), C/Josep Trueta s/n, 08017 Sant Cugat del Vallès, Spain; 3 ACTIUM Functional Anatomy Group, 08017 Barcelona, Spain; 4 Fundació Institut Universitari per a la Recerca a l’Atenció Primària de Salut Jordi Gol i Gurina, 08007 Barcelona, Spain; 5 Department of Nursing and Physiotherapy, Universitat de Lleida, 25003 Lleida, Spain; 6 Oxigena Fisioterapia, Calle N, 51, 50170 Mequinenza, Spain; 7 Hospital Universitari Santa Maria, Av. Alcalde Rovira Roure, 44, 25198 Lleida, Spain; 8 Department of Physical Therapy, Occupational Therapy, Physical Medicine and Rehabilitation, Universidad Rey Juan Carlos (URJC), 28922 Alcorcon, Spain

**Keywords:** COVID, exercise, performance, functional capacity, hospitalization

## Abstract

The worldwide pandemic caused by severe acute respiratory syndrome coronavirus 2 (SARS-CoV-2) virus has impacted all healthcare systems. One potential sequela experienced by hospitalized coronavirus disease 2019 (COVID-19) survivors includes muscle weakness with a reduction in strength and, consequently, a possible increase in frailty. The aim of this clinical trial was to evaluate the efficacy of adding an online therapeutic exercise program for 8 weeks to the medical prescriptions on functional variables in patients hospitalized due to COVID-19. A randomized controlled trial including 70 previously hospitalized COVID-19 survivors was conducted. Patients were randomly allocated to an experimental (n = 35) or control (n = 35) group. Both groups received regular prescriptions provided by their medical doctors. The experimental group also received a live online therapeutic exercise program for 8 weeks (3 sessions/week). Handgrip strength, gait speed, lower-extremity strength, balance, and frailty were assessed at baseline, at the end of the program, and one month after the end of the intervention. The repeated measures analysis of variance revealed significant Group*Time interactions for all the outcomes: (handgrip dominant: F = 17.395, *p* < 0.001, η^2^ = 0.24; handgrip non-dominant: F = 33.197, *p* < 0.001, η^2^ = 0.33; 4 m walk test (4WT): F = 13.039, *p* = 0.001, η^2^ = 0.16; short physical performance battery (SPPB): F = 26.421, *p* < 0.001, η^2^ = 0.28; the five chair-raise test (5CRT): F = 5.628, *p* = 0.004, η^2^ = 0.08; FRAIL scale: F = 11.249, *p* = 0.001, η^2^ = 0.14): patients in the experimental group experienced greater improvements in all outcomes than those assigned to the control group. This study revealed that the addition of an online exercise program for 8 weeks obtained greater improvements in handgrip strength, gait speed, lower-extremity strength, balance, and frailty in a sample of previously hospitalized COVID-19 survivors than application of just usual medical prescription.

## 1. Introduction

The global pandemic caused by severe acute respiratory syndrome coronavirus 2 (SARS-CoV-2), the agent causing the coronavirus disease 2019 (COVID-19), has collapsed healthcare systems around the world. Europe reported its first case in February 2020, and since then, the pandemic has quickly spread worldwide [1]. The consequences of SARS-CoV-2 have been extensively studied, and it has been identified that COVID-19 affects respiratory, cardiovascular, neurological, and gastrointestinal systems [2]. Data support that 35% of subjects who are hospitalized by COVID-19 require respiratory support. Additionally, 50% of patients requiring internal care unit (ICU) admission and, accordingly, requiring mechanical ventilation for more than five days develop generalized muscle weakness [3,4]. Evidence also shows a deterioration of functional capacity in patients who are discharged from either hospital wards [5,6] and ICU [6,7]. Although many patients with COVID-19 are hospitalized, the effects of COVID-19 hospitalization in functional repercussion has yet to be studied [8].

Exercise is an intervention aiming to improve human functional capacity [9]. Exercise has shown to be an effective tool for improving functionality and reducing disability in older adults [10] and in patients with heart and lung disease [11,12,13]. Zhu et al. [14] concluded that physical therapy will not only reduce the mortality rate of patients, hospital admission time, and medical expenses of COVID-19 patients, but it will also save medical resources and reduce personal and economic expenses and the probability of adverse social stability events, such as medical collapse. Therefore, physical therapy should be introduced into the mainstream treatment of patients with COVID-19 as early as possible. Lourenço et al. [15] explained the need for early mobilization and exercise of hospitalized patients with COVID-19 to prevent, reduce, and rehabilitate those consequences of the disease and post-intensive-care syndrome in patients with COVID-19. Exercise prescription for these patients should be implemented with caution, looking at each stage of the disease and the patients’ clinical condition to ensure that exercise is not too intense as to cause adverse effects but is sufficient to promote beneficial effects [15]. This aspect represents a challenge for clinicians, particularly in new diseases since there is no consensus on which exercise is the most appropriate [15]. A recent review concluded that programs consisting of resistance (e.g., 1–2 sets of 8–10 repetitions at 30–80% of 1RM) combined with aerobic (e.g., 5 to 30 min at moderate intensity) exercises may improve functional capacity and health-related quality of life in COVID-19 survivors [16]. In addition, different studies support the hypothesis that, whenever possible, it is advisable that survivors of COVID-19 perform strength exercises [17,18,19]. Accordingly, an exercise program could improve short-term consequences on functional capacity in post-hospitalization survivors of COVID-19 and may improve the overall health status of these patients. In fact, the pandemic has considerably limited face-to-face healthcare programs; therefore, online training (e.g., telerehabilitation) has been proposed as an effective and safe therapeutic modality [20]. In such a scenario, different studies corroborate that telerehabilitation is an important alternative to face-to-face exercise programs in selected patients since it decreases exposure and risk of infection between clinicians and patients [17,21,22,23]. No study has previously investigated the effects of adding a telerehabilitation program in people who had been hospitalized by COVID-19 on physical activity and frailty. Accordingly, the aim of this randomized clinical trial was to evaluate the efficacy of adding an online therapeutic exercise program to usual medical prescriptions on functional variables in individuals hospitalized due to COVID-19. Our hypothesis was that adding an 8-week online therapeutic exercise program to regular prescriptions would further improve functional capacity in previously hospitalized COVID-19 survivors.

## 2. Methods

### 2.1. Study Design

A parallel single-blinded, randomized controlled clinical trial (1:1) was conducted. The experimental group received usual general lifestyle recommendations from a medical doctor plus a structured therapeutic exercise on-line program, whereas the control group received just usual general lifestyle recommendations from the medical doctor. The duration of the interventions was 8 weeks, with a 1-month post-intervention follow-up. All interventions were applied during the first week after hospital discharge. This study was approved by the Ethics Committee of the Hospital Arnau de Villanova (CEIC-2468). The study followed current guidelines of the Declaration of Helsinki of the World Medical Association [24] as well as CONSORT [25] and TIDier [26] guidelines. Participants signed the written informed consent form before their inclusion in the study. The study was prospectively registered in clinicaltrials.gov (NCT04751630).

### 2.2. Participants

Ninety-three patients hospitalized due to SARS-CoV-2 infection were recruited at the Hospital Arnau de Villanova (Lleida, Spain) between June 2021 and February 2022. Inclusion criteria were adults (aged over 18 years old) hospitalized for at least three days because of COVID-19, with the ability to use a digital platform to carry out the online program with the healthcare provider. Exclusion criteria included previous diagnosis of any neurological or psychiatric disease, any contraindication for physical exercise, and lack of availability to perform the therapeutic exercise program three times a week [15]

### 2.3. Randomization and Masking

The randomization was performed by an external researcher who was not otherwise involved in the main trial and did not participate in analysis or interpretation of the results. Patients were randomly assigned to experimental or control group. Concealed allocation was performed with the sealed envelope program (London, UK) to generate the randomization sequence and assign a random number to each of the subjects. Individual and sequentially numbered index cards with the random assignment were prepared. The index cards were folded and placed in sealed opaque envelopes. Another researcher opened the envelope and proceeded with allocation. Treatment allocation was revealed to the participants after collection of baseline outcomes. The assessor obtaining follow-up data was blinded to the treatment allocation group.

### 2.4. Outcomes

The primary outcome of the study was handgrip strength, whereas secondary outcomes included gait speed, lower-extremity strength, balance, and frailty. These outcomes were assessed since they have been associated with risk of disability [27] and mortality [28,29,30]. All outcomes were assessed at the beginning of the study (T0), at the end of the intervention (T1), and one month after the intervention (T2) by an assessor blinded to the treatment allocation of the subjects.

Handgrip strength was assessed with the Jamar hand dynamometer (Sammons Preston, Inc., Bolingbrook, IL, USA). Participants were seated with the elbows flexed at 90° and performed a maximal handgrip strength for three seconds three times with each hand. The mean of the three attempts on each arm was used in the analysis. Evidence supports that handgrip strength is a good predictor of mortality and a marker of functional capacity [30,31]. This procedure has shown to have excellent reliability [32].

The short physical performance battery (SPPB) was used to assess the physical condition of participants [31,33,34]. This is a functional test battery widely used in primary care and research. It consists of three tests: balance test in feet-together position, semi-tandem and tandem for 10 s with eyes open; 4 m walking test; and five chair-raise test. Each result of the three tests has a numerical value from 0–4, which is summed to obtain a maximum overall score of 12 points [33,34]. Previous studies have established a cut-off score of 8 points for considering a person to be at risk for future negative events, such as falls, hospitalizations, sarcopenia, or frailty [30]. Test-retest reliability has been shown to be good to excellent (ICC 0.83–0.92), whereas inter-rater reliability has been found excellent (ICC 0.91) in elderly patients [35].

In addition, the 4 m walk (4MW) reflects the time (in seconds) that a patient needs to complete 4 m at a normal speed. The test was performed twice, and the shortest time was chosen for the analysis. Previous studies have established that a speed <0.8 m/s suggests that the person is at risk of future negative events, such as frailty, sarcopenia, falls, or hospitalization [31,36]. The reliability of the 4MW has been found to be excellent (ICC 0.96, 95%CI 0.94–0.98) [37].

The five chair-raise test (5CRT) reflects the time (in seconds) that a person needs to sit down and stand up five times from a chair with a backrest, without the help of the arms. It is included in the SPPB battery, but its score has individual value by itself. The test was performed twice, and the result with the shorter time was chosen for the analysis [31]. Previous studies have established that a cut-off point of 15 s reflects that an individual is at risk for future negative events, such as frailty, sarcopenia, falls, or hospitalization [36]. Test-retest reliability (ICC 0.83–0.92) and inter-rater reliability (ICC 0.91) have been shown to be excellent [35].

The FRAIL scale (score from 0 to 5) was used to identify the presence of frailty since it is one of the most widely used questionnaires in the literature [38,39]. This questionnaire allows screening people at a risk of being considered frail. It evaluates five items: fatigue, endurance, ambulation, comorbidity, and non-specific weight loss. Each item is scored with 1 point [38]. A person showing a FRAIL scale score ≥3 points is considered to be fragile, between 1–2 points suggest a pre-fragile individual, and 0 points is a non-fragile individual [40].

### 2.5. Interventions

Participants allocated to the control group received general lifestyle recommendations from their medical doctor [41]. These recommendations consisted of returning to their regular life progressively and starting to play sport (if previously practiced) and to work. In addition, they received a weekly video call to evaluate their current health status and to ensure the follow-up of this group. 

**Figure 1:**
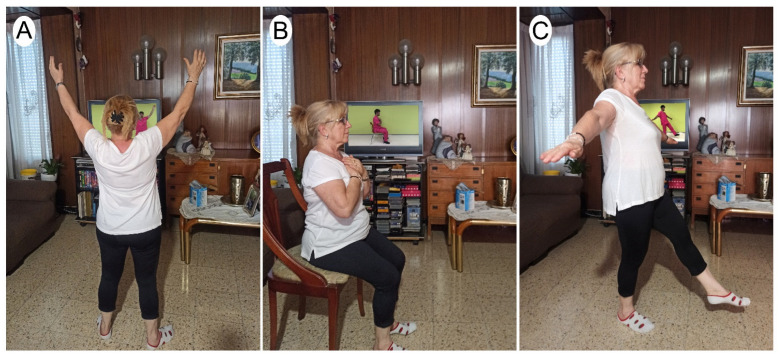
Three examples of exercises from ViviFail program. (**A**) Example of warm up; (**B**) example of strength exercise; (**C**) example equilibrium.

Participants allocated to the experimental group received the same prescriptions provided by their medical doctor as the control group. In addition, they received an 8-week live online exercise program, with a frequency of three sessions per week, for a total of 24 sessions. Each session consisted of 10 min of warm-up, 40 min of training, and 10 min of cool-down. A minimum of 20 sessions was established as necessary to be included in the study. All sessions were supervised by a physiotherapist expert in exercise prescription with more than 10 years of experience with the Meet Platform (Google). Exercises were extracted from the ViviFrail program [42,43] according to the physical condition of each participant (Figure 1).

The warm-up included breathing exercises, joint mobility, and walking exercises. Two weekly sessions focused on upper/lower-extremity strength and balance exercises. Bouts of three sets of 10–12 repetitions per exercise were performed. Main exercises used were double-legged squats. Help (using a chair) and weight (using a bottle or a box) were used as progressions. Knee extensions with and without weights or to raise a bottle with the hands as high as possible were strength exercises. Balance exercises consisted of single-legged and double-legged jumps. Progressions included doing exercises with eyes closed. The remaining session focused on interval training with work times of 20–40 s and rest times of 40–60 s. The intensity of the workouts ranged between 6 and 7 on the modified Borg scale (0–10), i.e., moderate to vigorous intensity [44]. Walking and going up and down stairs were the exercises most often used at this stage. The cool-down phase consisted of muscle group stretching, walking, and breathing exercises.

### 2.6. Study Procedure

After satisfying the eligibility criteria and agreeing to participate in the study, each subject was randomly assigned to the experimental or control group as described. During all evaluations and interventions, patients wore a pulse oximeter (Masimo MightySat *^®^* Rx) to monitor the safety of the procedure. The recommendations for training performed in people with COVID-19 were followed [15]. If any of the following conditions were met, it was immediate reason to stop exercise:

- Respiratory frequency < 40 breaths per minute;
- Heart rate < 120 beats/minute in resistance exercises and < 140 beats/minute in intervallic exercises;
- Sp02 < 90%.

Outcomes were assessed at the beginning of the study, at the end of the intervention, and one month after the intervention by an assessor blinded to the treatment allocation of the subjects.

### 2.7. Sample Size Calculation

The Gpower 3.1 software (Franz Faul, Universität Kiel, Kiel, Germany) was used for sample size calculation. It was calculated based on the Silva et al. [45] study, with an effect size of 0.68 in favor of the experimental group for upper-extremity strength (main outcome), a statistical power of 0.80 and an α = 0.05 value [46], a bilateral contrast, an allocation ratio N2/N1= 1, and an expected loss to follow-up of 25%. A required sample of 70 total participants was obtained, with 35 on each group.

### 2.8. Statistical Analysis

Statistical analysis was performed using the SPSSv.20 statistical package (IBM, Armonk, NU, USA). The Kolmogorov–Smirnov test was used to assess the normal distribution of the variables. Descriptive statistics were included for all variables, and quantitative variables were expressed as means and standard deviations, whereas qualitative or categorical variables were expressed as percentages. Baseline between-groups differences were compared with independent Student’s *t*-test (normal distribution) or Mann–Whitney U-test (if non-normal distribution). 

A linear mixed model (ANOVA) with time (baseline, post-intervention, one month after) and group (experimental, control) was conducted for determine changes in the outcomes. Effect sizes (ES) were calculated using eta squared (η^2^). An effect size >0.14 was considered as large; around 0.06 as medium; and <0.01 small [47]. If significant differences existed, the Bonferroni post hoc correction was performed to determine between-groups differences for each period (T0–T1; T1–T2; T0–T2). Statistical analysis was performed by intention-to-treat. The significance level was set at *p* < 0.05.

## 3. Results

**Figure 2:**
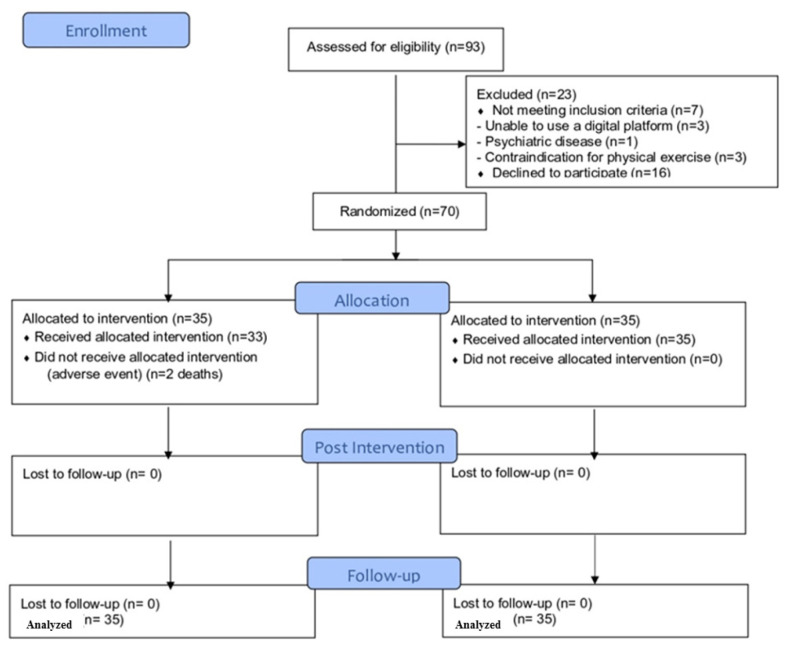
Flow diagram of participants during the study.

Between June 2021 and February 2022, 93 patients hospitalized due to COVID-19 were screened for eligibility criteria. Seventy patients (47 men, 23 women) satisfied all eligibility criteria and agreed to participate. Subjects were randomly assigned to each group (n = 35) and received the assigned treatment, and their data were analyzed by intention-to-treat. Figure 2 shows the flow diagram of the study. Both groups were comparable at baseline (Table 1).

### 3.1. Primary Outcome

The repeated measures ANOVA revealed significant Group*Time interactions in both dominant (F = 17.395, *p* < 0.001, η^2^ = 0.24) and non-dominant (F = 33.197, *p* < 0.001, η^2^ = 0.33) handgrip: patients assigned to the experimental group experienced greater improvements (with large effect sizes) than those assigned to the control group (Table 2).

The Bonferroni post hoc analysis identified that the experimental group reported large clinical improvement at T0–T1 and T0–T2 (all, *p* < 0.001), whereas the control group did not show any significant change (Table 2).

### 3.2. Secondary Outcomes 

The repeated measures ANOVA revealed significant Group*Time interactions for secondary outcomes (4MW: F = 13.039, *p* = 0.001, η^2^ = 0.16; SPPB: F = 26.421, *p* < 0.001, η^2^ = 0.28; 5CRT: F = 5.628, *p* = 0.004, η^2^ = 0.08; FRAIL scale: F = 11.249, *p* = 0.001, η^2^ = 0.14): individuals in the experimental group experienced greater improvements (moderate to large effect sizes) in all secondary outcomes than those assigned to the control group (Table 2).

The Bonferroni post hoc analysis identified that the experimental group obtained large clinical improvements in secondary outcomes at T0–T1 and T0–T2 (all, *p* < 0.001), whereas the control group showed small to medium improvements in 4MW at T0–T1 (*p* = 0.005), 5CRT at T0–T1 and T0–T2 (*p* < 0.01), and FRAIL scale at T0–T2 (*p* < 0.001).

## 4. Discussion

This clinical trial has shown that adding an online exercise program to the usual medical prescriptions for 8 weeks after hospital discharge improved different functional variables in hospitalized patients who survived COVID-19. Those improvements were clinically relevant in all outcomes in favor of the experimental group.

We found a significant improvement on handgrip strength only in the experimental group at the end of the intervention and at the end of the last follow-up, with large effect sizes. Nambi et al. [48] evaluated changes in handgrip strength in COVID-19 survivors. In their study, patients received high-intensity aerobic training or resistance training for 8 weeks, 4 days a week. The authors observed that, at one-month follow-up, there was no significant improvement in handgrip strength in either of the two training groups, but some changes were observed at the second month [48]. Other studies showed that handgrip strength is strongly associated with functional training, lower-extremity strength, or balance [8,49]. Despite these discrepancies, it appears that when training is maintained, and especially when it focuses on the lower and upper extremities, it can cause significant improvements in handgrip strength and functional capacity, decreased falls, and decreased mortality [50,51,52]. In spite of the fact that there are no similar studies in COVID-19 survivors, there are similar studies in frail older adults. Casas-Herrero et al. [52] observed a significant improvement in handgrip strength after the application of a multicomponent program for 12 weeks in comparison with a control group. Sadjapong et al. [53] found significant improvements in handgrip strength after applying a 24-week multicomponent training program (12 weeks face-to-face plus 12 weeks home-based) when compared with a control group at a 12-week follow-up but not at 24-week follow-up. Finally, Suikkanen et al. [54] did not find between-groups differences in handgrip strength.

There are different studies that evaluate the SPPB in patients with COVID-19 [41,42,43,44]; however, there is only one study assessing changes after an exercise intervention [45]. In this study, without control group, the authors observed that after a 7-day multicomponent training, the SPPB improved an average of 3.5 points [45], which is in agreement with our study. In fact, we found significant differences in favor of the experimental group at the end of treatment and at 1-month follow-up. Scores equal to or below 8 points were related to frailty or sarcopenia [46]. At the beginning of the study, both groups (control: 8.2; experimental: 7.6) were below these values. At the end of the intervention and at the 1-month follow-up, the experimental but not the control group reached normative values of 10.2 and 10.5, respectively. The improvement of functional capacities throughout exercise and its relationship with the improvement of cardiorespiratory capacities has been widely studied in other adult populations [47].

Balance was one of the items assessed by the SPPB. In our study, part of the exercise program focused on balance training and could justify the improvement in this variable. Chikhanie et al. [48] performed breathing training in hospitalized COVID-19 survivors. A small part of this training consisted of balance exercises. These authors observed an improvement in these patients very similar to those reported in our study. However, Udina et al. [45] did not find significant improvements after a multicomponent training program in COVID-19 survivors.

In relation to gait speed variables, we found statistically significant differences in favor of the experimental group. Other studies found improvements of half a second in the 4MWT in these patients after applying exercise training sessions [45]. These data are lower than those obtained in our study, in which the experimental group achieved a 2.5 s improvement in running speed. It is possible that these differences are due to the fact that in our study, the training was applied for 8 weeks compared to one week in Udina et al. [45].

We also observed that the experimental group improved in speed on the 5CRT when compared with the control group. Our data are superior to previous studies [45], suggesting that the duration of the exercise intervention can be a key point to obtain positive results. At one-month follow-up, we observed improvement in both groups on the FRAIL scale; however, large improvements were only seen in the experimental group. These data suggest that an exercise program provides faster and more beneficial rehabilitation for previously hospitalized COVID-19 survivors. International exercise recommendations suggest that training improves frailty and thus reduces falls and mortality, leading to a better health status for the patient 49].

The main limitation of this study is that due to the pandemic conditions in which they were conducted, part of the training was conducted telemetrically, losing or limiting the possibility of the potential benefit of social interaction. Despite minimizing all possible biases, it is likely that a full group face-to-face intervention would have led to better outcomes, as recommended by clinical guidelines [49]. In addition, we did not collect potential hospitalization data, such as treatment received. 

## 5. Conclusions

An online exercise program combined with medical prescriptions for 8 weeks exerted greater improvements in handgrip, 4MW, SPPB, 5CRT, and FRAIL scale in individuals who had been hospitalized by COVID-19 compared to usual medical prescription. Tele-rehabilitation programs could help to manage people at risk after COVID-19 hospitalization.

## Figures and Tables

**Table 1:** Baseline features of both groups.

	Experimental Group	Control Group
	Mean ± SD	Mean ± SD
Age (years)	49.5 ± 13.7	55.1 ± 20.9
Sex		
Men, n (%)	24 (68.6%)	23 (65.7%)
Women, n (%)	11 (31.4%)	12 (34.3%)
Height (m)	1.71 ± 0.1	1.7 ± 0.3
Weight (kg)	87.1 ± 19.2	80.1 ± 13.9
BMI (Kg/m^2^)	29.7 ± 5.1	27.2 ± 4.4
Dominance		
Right, n (%)	26 (74.3%)	31 (88.6%)
Left, n (%)	9 (25.7%)	4 (11.4%)
Days of physical activity per week before COVID-19	3.0 ± 2.8	3.2 ± 3.1
Sitting hours	5.6 ± 3.1	6.5 ± 3.2
Days of hospitalization	14.3 ± 14.8	9.5 ± 8.1

**Abbreviations:** SD, standard deviation.

**Table 2:** Descriptive data and effect sizes for dependent variables.

		Baseline T0	Post-Intervention T1	Follow-up T2	Difference between T0–T1		Difference between T1–T2		Difference between T0–T2	
		Mean ± SD	Mean ± SD	Mean ± SD	Mean (95% CI)	η^2^	Mean (95% CI)	η^2^	Mean (95% CI)	η^2^
Control Group	Handgrip (Kg)									
	Dominant	32.1 ± 11.8	32.3 ± 15.5	32.5 ± 15.5	0.2 (−4.4; 4.9)	0.00	0.2 (−1.4; 1.8)	0.00	0.4 (−4.4; 5.2)	0.00
	Non-dominant	29.9 ± 10.8	28.2 ± 15.0	27.7 ± 15.1	−1.7 (−5.6; 2.3)	0.00	−0.5 (−1.9; 1.0)	0.00	−2.2 (−6.2; 1.9)	0.01
	4 m walk (sg)	6.6 ± 3.1	5.5 ± 3.4	5.9 ± 3.4	−1.1 (−1.9; 0.3)	0.03	0.4 (−0.4; 0.7)	0.00	−0.7 (−1.6; 0.0)	0.01
	5CRT (sg)	18.6 ± 13.0	15.1 ± 10.4	14.8 ± 10.1	−3.5 (−5.8; −1.3)	0.02	−0.3 (−0.9; 0.4)	0.00	−3.8 (−6.1; −1.5)	0.03
	Total SPPB (0–12)	8.2 ± 2.5	8.7 ± 3.4	8.6 ± 3.3	0.5 (−0.5; 1.5)	0.01	−0.1 (−0.5; 0.2)	0.00	0.4 (−0.7; 1.4)	0.01
	FRAIL scale (5–0)	2.7 ± 1.4	2.1 ± 1.7	1.7 ± 1.7	−0.6 (−1.3; 0.1)	0.03	−0.4 (−0.9; 0.8)	0.03	−1.0 (−1.7; −0.4)	0.10
Experimental Group	Handgrip (Kg)									
	Dominant	25.2 ± 13.3	35.5 ± 9.3	37.1 ± 10.2	10.3 (−5.7; 15.0)	0.17	1.6 (0.1; 3.1)	0.01	11.9 (7.1; 16.7)	0.20
	Non-dominant	26.6 ± 13.2	36.7 ± 11.1	37.9 ± 11.4	10.1 (6.2; 14.0)	0.15	1.2 (−0.3; 2.6)	0.00	11.3 (7.2; 15.3)	0.18
	4 m walk (sg)	6.5 ± 1.8	4.4 ± 1.5	4.0 ± 1.2	−2.2 (−2.9; −1.4)	0.26	−0.4 (−0.7; 0.0)	0.02	−2.5 (−3.2; −1.6)	0.32
	5CRT (sg)	19.5 ± 7.5	13.1 ± 6.0	12.4 ± 5.8	−6.4 (−8.7; −4.2)	0.15	−0.7 (−1.3; −0.1)	0.00	−7.1 (−9,5; −4.8)	0.18
	Total SPPB (0–12)	7.5 ± 1.6	10.6 ± 1.8	10.9 ± 1.7	3.2 (2.2; 4.2)	0.26	0.3 (−0.1; 0.7)	0.00	3.4 (2.4; 4.4)	0.30
	FRAIL scale (5–0)	2.9 ± 1.0	2.0 ± 1.2	0.6 ± 0.8	−0.9 (−1.6; −0.2)	0.14	−1.4 (−1.9; 0.9)	0.18	−2.3 (−2.9; −1.6)	0.46

## Data Availability

Not applicable.
